# Mixture of prompts learning for vision-language models

**DOI:** 10.3389/frai.2025.1580973

**Published:** 2025-06-10

**Authors:** Yu Du, Tong Niu, Rong Zhao

**Affiliations:** ^1^Center for Brain-Inspired Computing Research (CBICR), Tsinghua University, Beijing, China; ^2^Optical Memory National Engineering Research Center, Tsinghua University, Beijing, China; ^3^Department of Precision Instrument, Tsinghua University, Beijing, China; ^4^IDG/McGovern Institute for Brain Research, Tsinghua University, Beijing, China; ^5^CETC Haikang Group-Brain Inspired Computing Joint Research Center, Beijing, China

**Keywords:** prompt learning, vision-language model, mixture-of-experts, multi-modal, few-shot classification

## Abstract

As powerful pre-trained vision-language models (VLMs) like CLIP gain prominence, numerous studies have attempted to combine VLMs for downstream tasks. Among these, prompt learning has been validated as an effective method for adapting to new tasks, which only requires a small number of parameters. However, current prompt learning methods face two challenges: first, a single soft prompt struggles to capture the diverse styles and patterns within a dataset; second, fine-tuning soft prompts is prone to overfitting. To address these challenges, we propose a mixture-of-prompts learning method incorporating a routing module. This module is able to capture a dataset's varied styles and dynamically select the most suitable prompts for each instance. Additionally, we introduce a novel gating mechanism to ensure the router selects prompts based on their similarity to hard prompt templates, which both retains knowledge from hard prompts and improves selection accuracy. We also implement semantically grouped text-level supervision, initializing each soft prompt with the token embeddings of manually designed templates from its group and applying a contrastive loss between the resulted text feature and hard prompt encoded text feature. This supervision ensures that the text features derived from soft prompts remain close to those from their corresponding hard prompts, preserving initial knowledge and mitigating overfitting. Our method has been validated on 11 datasets, demonstrating evident improvements in few-shot learning, domain generalization, and base-to-new generalization scenarios compared to existing baselines. Our approach establishes that multi-prompt specialization with knowledge-preserving routing effectively bridges the adaptability-generalization tradeoff in VLM deployment. The code will be available at https://github.com/dyabel/mocoop.

## 1 Introduction

Recently, pre-trained vision-language models (VLMs), such as CLIP (Radford et al., 2021), have gained increasing prominence. Numerous studies have explored their applications in various downstream tasks, including image classification (Zhou et al., 2022b), visual question answering (VQA) (Eslami et al., 2021), and cross-modal generation (Crowson et al., 2022; Saharia et al., 2022). Among these, prompt learning has emerged as an effective approach for enhancing performance on downstream tasks by optimizing the prompts fed into the model. This method achieves significant improvements without requiring large-scale fine-tuning of the entire model.

In the context of image classification, for example, a prompt essentially serves as a template that can be positioned before, after, or around the class name. Traditionally, manually designed text templates, known as hard prompts, were employed during CLIP's training to guide the model in associating textual descriptions with visual content. Prompt learning builds upon this approach by replacing these fixed text templates with learnable continuous vectors, referred to as soft prompts. By fine-tuning these vectors with a small number of samples, soft prompts can significantly improve performance on downstream tasks, offering a more flexible and efficient alternative to hard prompts.

A foundational contribution to this area is the Context Optimization (CoOp) model (Zhou et al., 2022b), which optimizes prompt contexts to enhance the performance of models like CLIP (Radford et al., 2021) in few-shot learning scenarios.

Building upon this, researchers have proposed vision prompts (Zang et al., 2022; Khattak et al., 2023), where learnable vectors are appended to the inputs of a vision encoder, akin to how text prompts are used in language models. While this approach demonstrates significant performance improvements, it comes at the expense of increased computational costs. In this paper, we focus exclusively on text-based prompts, and our methodology could be extended to incorporate vision prompts in future work.

Despite the success of prompt learning, many methods face a trade-off between classification accuracy and robustness. Improper fine-tuning of soft prompts can degrade performance, causing the model to underperform compared to the zero-shot capabilities of the original Vision-Language Models (VLMs) (Radford et al., 2021; Zhou et al., 2022b). This issue arises primarily due to over-training on base classes, which leads to catastrophic forgetting of domain-general knowledge (Zhu et al., 2023).

To address this, several approaches have sought to constrain the optimization of soft prompts by utilizing features derived from manual templates (Zhou et al., 2022a; Yao et al., 2023; Bulat and Tzimiropoulos, 2022; Zhu et al., 2023). These approaches commonly restrict gradient updates or employ knowledge distillation techniques.

Among these methods, ProGrad (Zhu et al., 2023) mitigates the issue of prompt tuning that forgets general knowledge in VLMs by updating prompts only when their gradients align with the “general direction,” as represented by the gradient of the KL divergence loss from a predefined prompt. Additionally, KgCoOp (Yao et al., 2023) reduces the discrepancy between textual embeddings generated by learned prompts and those derived from hand-crafted prompts. Inspired by these approaches, our work distills knowledge from original text features into each expert soft prompt. Furthermore, we introduce gating regularization to distill prior knowledge from discrete text templates into the router, thereby improving prompt selection accuracy.

**Figure 1:**
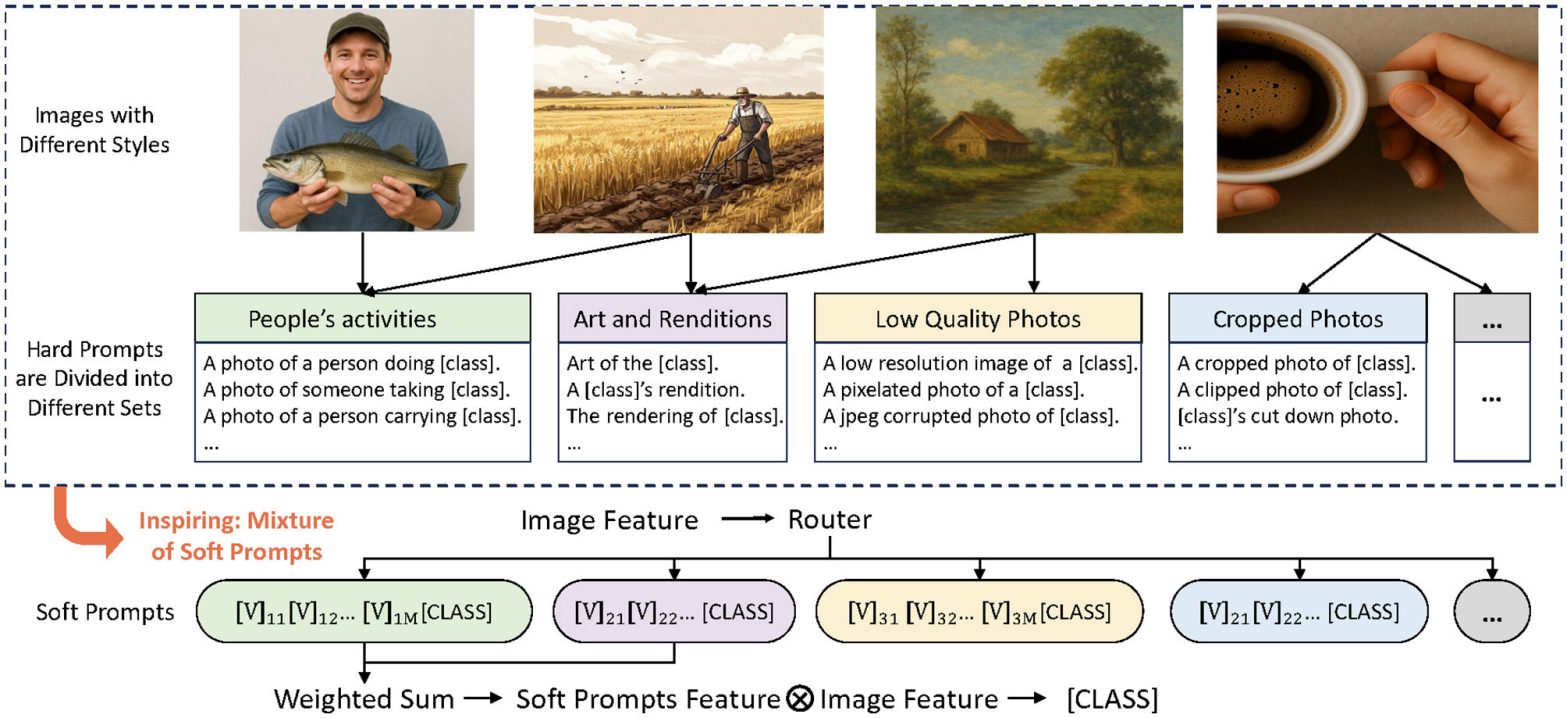
For the image classification task based on CLIP (Radford et al., 2021), hard templates can be grouped into different sets based on the contexts and patterns they describe in the images (e.g., varying contents within differently colored blocks). Different images are usually present with different context styles and a single image may simultaneously exhibit multiple styles. Traditionally, only one soft prompt is used to represent all images, which limits adaptability. In contrast, our method utilizes multiple soft prompts, with each soft prompt representing a distinct context. A routing module dynamically selects the most suitable prompts for each image. By accounting for different styles, this approach more effectively bridges the gap between visual and textual features.

However, these methods generally overlook the diverse context styles present in different images. A single soft prompt may fail to capture multiple styles. As illustrated in Figure 1, different instances in the same dataset may align better with distinct prompts. Thus, using multiple prompts is more effective in representing these variations.

From this motivation, we propose a mixture-of-prompts learning method. This method integrates a routing module that dynamically selects the most suitable prompts for each instance. The selected prompts are encoded by a text encoder to generate multiple sets of class text features. These features are then weighted and averaged to produce a final set of class text features, which are compared with image features to compute similarities. Conceptually, this process can be viewed as selecting the most compatible style prompts for each instance, thereby enhancing the system's adaptability and performance.

To improve the router's effectiveness, we introduce a hard-prompt-guided gating loss. This loss function ensures that the router selects the soft prompts whose corresponding hard prompt encoded text features align most closely with the corresponding image features. By incorporating this mechanism, we distill the knowledge embedded in the hard prompt templates into the router, encouraging it to make more accurate and contextually relevant prompt selections.

Additionally, to mitigate overfitting, we propose semantically grouped text-level supervision. Each soft prompt is associated with a set of manually designed templates (hard prompts) that share relatively similar semantic contexts. The token embeddings of one template from each set are used to initialize the corresponding soft prompt. During training, the text features generated by the text encoder for each soft prompt are constrained to remain close to the text features of their associated hard prompts. This ensures that the initial knowledge from the manual text templates is preserved and effectively integrated into the soft prompts.

We validated our method across 11 datasets, evaluating its performance in few-shot learning, and base-to-new generalization. Our approach consistently outperformed existing baselines, demonstrating improvements in adaptability and generalization. Furthermore, we conducted extensive ablation studies to assess the contribution of individual components, confirming their roles in driving the observed performance gains.

In summary, our contributions are as follows:

- We propose a mixture-of-prompts learning method that incorporates a routing module to dynamically select the most suitable prompts for each instance.
- We introduce a hard prompt-guided gating loss, which ensures that the router selects prompts based on their similarity to hard prompt templates, thereby improving selection accuracy.
- We implement semantically grouped text-level supervision to preserve the initial knowledge from manual text templates and mitigate overfitting.
- We validate our method across 11 datasets, demonstrating improvements in few-shot learning and base-to-new generalization scenarios compared to existing baselines.

## 2 Method

### 2.1 Overview

**Figure 2:**
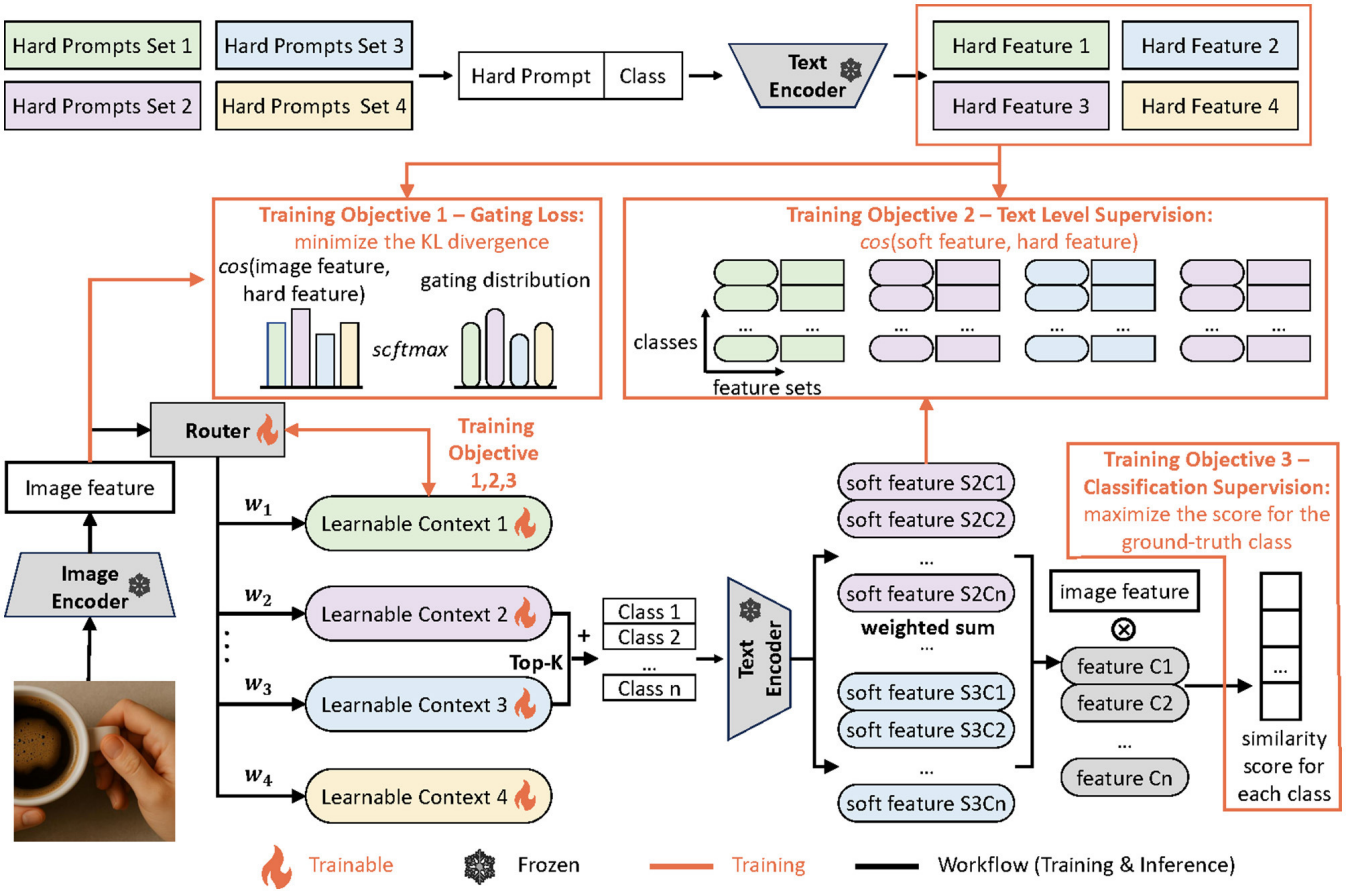
Overview of MoCoOp. The orange lines signify the extra flow for training while the black lines are shared by training and inference. During inference, two soft prompts with the highest probabilities are selected and combined with the available classes for text encoding. The resulting text features are averaged and used for classification. During training, the hard prompt guided routing and semantically grouped text level supervision are introduced to supervise the router and soft prompts respectively.

As illustrated in Figure 2, our MoCoOp framework consists of three key components: (1) a router module that dynamically selects the most suitable prompts for each image, (2) multiple learnable soft prompts that capture different context styles, and (3) a mechanism for combining these prompts to generate effective text features.

During inference, an input image is first processed by the CLIP image encoder to extract its visual features. These features are fed into the router module, which calculates selection probabilities for each available soft prompt. The router then selects the top-k prompts with the highest probabilities.

Each selected soft prompt is concatenated with all class names and processed by the CLIP text encoder, generating k sets of class text features. These sets are then combined using a weighted average, with weights determined by the normalized probabilities from the router's gating distribution. This weighted combination produces a single, contextually enriched set of class text features that better aligns with the image content. Finally, the cosine similarity between this combined text feature and the image feature determines the classification logits.

By activating only k soft prompts at inference time (where k is typically set to 2 in our experiments), our approach maintains computational efficiency while leveraging the benefits of multiple specialized prompts. This design effectively balances the trade-off between model expressiveness and inference cost.

During training, there are three parts of gradient flow. First, we apply a cross-entropy loss between the final classification probabilities and the ground truth labels. Second, for the router, we calculate the similarity between the image feature and the representative text features from each hard prompt template set which are obtained by averaging across all classes and all templates in the set. These similarities serve as a reference distribution for the router's gating mechanism. Next, we use a KL divergence loss to align the router's gating distribution with this reference distribution. Finally, for the soft prompts, we apply another cross-entropy loss to ensure that the text features generated by the soft prompts closely match the corresponding class features produced by the associated hard prompts. By aligning the router's gating distribution with the reference distribution and ensuring consistency between soft and hard prompts, the model learns to both specialize and generalize effectively for accurate classification.

### 2.2 Preliminary of CoOp

Here we give a brief introduction of CoOp (Zhou et al., 2022b), the pioneering work in prompt learning of VLMs.

Context Optimization (CoOp) is a method for adapting vision-language models like CLIP to downstream tasks with limited labeled data. CLIP was originally trained using text templates such as “a photo of a [CLASS]” to generate text features. Instead of using these fixed templates, CoOp replaces them with learnable vectors while keeping the pre-trained CLIP encoders frozen. These learnable vectors, called soft prompts, can capture task-specific knowledge during fine-tuning and have proven more effective than manually designed prompts. CoOp only requires optimizing a small number of parameters (the soft prompts), making it particularly efficient for few-shot learning scenarios.

**Notation:**

First, here are some notations used in prompt learning of VLMs.

- **x**: Input image
- **p**: Text prompt
- *f*_img_: CLIP image encoder
- *f*_txt_: CLIP text encoder
- **h**_*x*_ = *f*_img_(**x**): Encoded image feature
- **h**_*p*_ = *f*_txt_(**p**): Encoded text feature
- **SP**: Soft prompt vectors (also referred to as context vectors) (learnable parameters)

#### 2.2.1 Prompt representation

The text prompt **p** is represented as a sequence of tokens, including learnable soft prompt tokens and a class token.


p=[SP, CLASS]

The soft prompt tokens can also be placed after or around the class token.

#### 2.2.2 Context

- The soft prompt is learnable vectors **SP** = [**sp**_1_, **sp**_2_, …, **sp**_*M*_], where ${\text{s}\text{p}}_{i}\in {\mathbb{R}}^{d}$ and *M* is the number of soft prompt tokens.
- All classes share the same soft prompt **SP** or each class *c* has its own soft prompt **SP**_*c*_.

#### 2.2.3 Training objective

Given a dataset with images {**x**_*i*_} and corresponding labels {*y*_*i*_}, the goal is to find the optimal soft prompt vectors **SP** (or **SP**_*c*_ for class-specific soft prompts) by minimizing the cross-entropy loss:


$$L=-\underset{i}{\sum }\log\frac{\exp\left(\text{sim}\left({\text{h}}_{x}^{i},{\text{h}}_{p}^{y_{i}}\right)/\tau \right)}{\underset{c}{\sum }\exp\left(\text{sim}\left({\text{h}}_{x}^{i},{\text{h}}_{p}^{c}\right)/\tau \right)}$$

where

- ${\text{h}}_{x}^{i}=f_{\text{img}}\left({\text{x}}_{i}\right)$ is the image feature for image *i*.
- ${\text{h}}_{p}^{c}=f_{\text{txt}}\left(\left[\text{S}\text{P},{\text{CLASS}}_{c}\right]\right)$ is the text feature for class *c*.
- sim(·, ·) denotes a similarity function, such as cosine similarity.
- τ is the temperature.

#### 2.2.4 Optimization

The soft prompt vectors **SP** are updated through backpropagation to minimize the loss L, while keeping the pre-trained parameters of *f*_img_ and *f*_txt_ fixed.

In summary, CoOp involves learning optimal soft prompt vectors **SP** for text prompts, which are used to synthesize classification weights for downstream tasks. This process automates prompt engineering and enhances the adaptability and performance of vision-language models like CLIP (Radford et al., 2021) on various image recognition tasks.

### 2.3 Mixture of prompts learning

The essential idea of this work is to extend the concept of context vectors in CoOp to a mixture-of-experts framework. While CoOp learns a single context vector shared across all instances, our approach learns multiple soft prompts (which are essentially context vectors) and dynamically selects the most suitable ones for each input.

In our framework, the router selects the top K soft prompts for each input image. These selected soft prompts are concatenated with the class names and encoded by the text encoder to obtain several sets of class features:


(1) $$\begin{array}{l} {\text{h}}_{p_{k}}=f_{\text{txt}}\left(\left[{\text{S}\text{P}}_{k},\text{ CLASS}\right]\right) \end{array}$$

for *k* = 1, 2, …, *K*, where **SP**_*k*_ is the soft prompt for the *k*-th selected expert.

The features are then weighted and averaged to produce the final set of class features:


(2) $$\begin{array}{l} {\text{h}}_{p}=\overset{K}{\underset{k=1}{\sum }}w_{\text{router}}^{k}f_{\text{txt}}\left(\left[{\text{S}\text{P}}_{k},\text{ CLASS}\right]\right) \end{array}$$

where $w_{\text{router}}^{k}$ are the weights assigned to each prompt feature. A cross entropy loss is utilized to optimize these prompts:


(3) $$\begin{array}{l} L_{\text{cls}}=-\underset{i}{\sum }\log\frac{\exp\left(\text{cos}\left({\text{h}}_{x}^{i},{\text{h}}_{p}^{y_{i}}\right)/\tau \right)}{\underset{c\in C}{\sum }\exp\left(\text{cos}\left({\text{h}}_{x}^{i},{\text{h}}_{p}^{c}\right)/\tau \right)} \end{array}$$

where C is the set of all classes.

### 2.4 Hard prompt guided routing

Given *G* sets of hard prompts (*I*_1_, *I*_2_, …*I*_*G*_), each concatenated with every class and encoded through the CLIP text encoder, we obtain *G* sets of hard text features for all classes. Specifically, for a hard prompt concatenated with a specific CLASS_*c*_, the corresponding hard text features can be similarly obtained using the CLIP text encoder, resulting in:


(4) $$\begin{array}{l} {\text{h}}_{c}^{\text{hard}}=f_{\text{txt}}\left(\left[\text{hard\_prompt},{\text{ CLASS}}_{c}\right]\right) \end{array}$$

where *c* denotes the specific class.

These hard text features are then averaged to generate *G* group text features, each representing one of the *G* groups. Specifically, the group text feature **h**_*g*_ for the *g*-th group is computed by averaging the hard text features for all classes and all templates within that group as:


(5) $$\begin{array}{l} {\text{h}}_{g}^{\text{hard}}=\frac{1}{\left|I_{g}\right|}\underset{i\in I_{g}}{\sum }\frac{1}{\left|C\right|}\underset{c\in C}{\sum }{\text{h}}_{i,c}^{\text{hard}} \end{array}$$

where C represents the set of all classes, and **h**_*i, c*_ represents the i-th hard text feature for class *c* in the *g*-th group.

The cosine similarity between the image feature **v** and each group's text feature, is calculated. The hard prompt guided gating distribution *W*_hard_ is then derived by applying the softmax function to these similarity scores, expressed as:


(6) $$\begin{array}{l} W_{\text{hard}}=\text{Softmax }\left(\begin{array}{c} \cos\left({\text{h}}_{1}^{\text{hard}},\text{v}\right)\\ \cos\left({\text{h}}_{2}^{\text{hard}},\text{v}\right)\\ \vdots \\ \cos\left({\text{h}}_{G}^{\text{hard}},\text{v}\right) \end{array}\right) \end{array}$$

The router's output gating distribution is denoted by *W*_router_. To ensure coherence between the two distributions, KL divergence is employed as a constraint, with the loss function defined as:


(7) $$\begin{array}{l} L_{\text{router}}=D_{\text{KL}}\left(W_{\text{router}}∥W_{\text{hard}}\right) \end{array}$$

### 2.5 Semantically grouped text level supervision

To mitigating the overfitting issue, we introduce semantically grouped text level supervision. The semantically similar groups of hard prompts are primarily manually curated based on their contextual and semantic relationships, though GPT-4 could also be employed to assist with automated grouping. We grouped templates that describe similar visual aspects or characteristics together through a combination of domain knowledge analysis and semantic similarity assessment. This process could be enhanced using large language models like GPT-4 to analyze prompt semantics and automatically cluster them into coherent groups. For example, prompts like “a photo of a {class}” and “an image showing a {class}” naturally form a group through their shared general descriptive patterns, while prompts like “a close-up photo of a {class}” and “a detailed view of a {class}” cluster into a detail-oriented group—relationships that could be automatically detected using GPT-4's semantic understanding capabilities.

The hard prompts are semantically grouped into G sets *I*_1_, *I*_2_, …*I*_*G*_. For each learnable soft prompt ${\text{t}}_{g}^{s}$ and its corresponding hard prompt group *I*_*g*_, the probability of a class y filled in this soft prompt being classified as its labeled class *y* is given by:


(8) $$\begin{array}{l} P\left(y|{\text{S}\text{P}}_{g}\right)=\frac{1}{\left|I_{g}\right|}\underset{i\in I_{g}}{\sum }\;P_{i}\left(y|{\text{S}\text{P}}_{g}\right)\\ P_{i}\left(y|{\text{S}\text{P}}_{g}\right)=\frac{\exp\left(\cos\left({\text{H}\text{P}}_{i}^{y},f_{\text{txt}}\left(\left[{\text{S}\text{P}}_{g},y\right]\right)\right)/\tau \right)}{\underset{c\in C}{\sum }\exp\left(\cos\left({\text{H}\text{P}}_{i}^{c},f_{\text{txt}}\left(\left[{\text{S}\text{P}}_{g},c\right]\right)\right)/\tau \right)} \end{array}$$

where *P*_*i*_(*y*|**SP**_*g*_) is the probability of class *y* being correctly classified when using the *g*-th soft prompt **SP**_*g*_ and compared with the *i*-th hard template in group *I*_*g*_. Here, ${\text{H}\text{P}}_{i}^{y}$ represents the hard prompt text feature for class *y* using the *i*-th template, *f*_txt_([**SP**_*g*_, *y*]) is the text feature obtained by concatenating the *g*-th soft prompt with class *y*, cos(·, ·) denotes the cosine similarity, τ is a temperature parameter, and C is the complete set of classes.

Next, we use the cross-entropy loss to minimize the distance between the encoded learnable soft prompts and the manually defined text prompts in the encoded space. The loss function can be expressed as:


(9) $$\begin{array}{l} L_{\text{text}}=-\frac{1}{G}\overset{G}{\underset{g=1}{\sum }}\underset{c\in C}{\sum }\frac{1}{\left|C\right|}\log P\left(c|{\text{S}\text{P}}_{g}\right) \end{array}$$

The overall training objective is


(10) $$\begin{array}{l} L=L_{\text{cls}}+\lambda_{1}L_{\text{router}}+\lambda_{2}L_{\text{text}} \end{array}$$

Where λ_1_ and λ_2_ are weights that balance the importance of each loss term.

## 3 Experiment

### 3.1 Settings

We conduct experiments under two settings: base-to-new generalization and few-shot learning. For base-to-new generalization, we split the classes into two groups, one as base classes and the other as new classes. We train on the base class, and test on both the base classes and new classes. For few-shot learning, we train and test on all classes. The few-shot capability reflects the method's fitting ability, while base-to-new generalization can measure the model's robustness.

### 3.2 Implementation and training details

For each expert, we use different context positions depending on the handcrafted template object used to initialize it. We used 4 to 20 experts. The number of experts and corresponding templates varies for datasets. For selecting the number of experts, we recommend starting with a smaller number (4–8) for datasets with clearly defined visual categories and increasing to 10–20 for datasets with diverse visual characteristics. The optimal number can be determined through validation performance. As a general guideline, specialized datasets (e.g., aircraft, flowers) work well with 4–8 experts, general datasets with diverse categories (e.g., ImageNet) benefit from 15 to 20 experts, and datasets with moderate diversity perform best with 8–12 experts. These guidelines are based on our empirical observations during experimentation and provide a reasonable starting point for practitioners applying our method to new datasets. For example, for FGVCAircraft, we use the template “a photo of a {}, a type of aircraft.” For the OxfordFlowers dataset, we use “a photo of a {}, a type of flower.” Generally, a custom template for each dataset is combined with some general templates like “a photo of a ”. Since ImageNet covers a wide range of categories, we use 20 groups of templates. Specific templates can be found in the Supplementary material.

Regarding the router architecture, we employ a simple design consisting of a single linear layer. The input to the router is the image feature from CLIP's image encoder (dimension 512), and the output is a distribution over the available prompt groups (dimension = number of experts). We intentionally kept the router architecture simple to maintain computational efficiency while still providing effective routing capabilities. This linear layer directly projects the image features to the number of experts.

Following the standard comparison setup in most recent works, we use different backbone architectures for different experimental settings: ViT-B/16 for base-to-new generalization and ResNet50 for few-shot learning. Specifically, we use the publicly available CLIP models (https://github.com/openai/CLIP). The resolution of CLIP's feature map is 14 × 14 for CLIP-ViT-B/16. The λ_1_ and λ_2_ are set as 1 and 5, respectively. The τ in Equations 3, 8 is set to 0.07. Our training schedule is consistent with CoOp (Zhou et al., 2022b), and both training and testing are conducted on four NVIDIA GeForce RTX 3090 GPUs.

### 3.3 Evaluation metrics and baselines

For few-shot experiments, we use top-1 accuracy. For base-to-new generalization, we evaluate by **base** class accuracy, **new** class accuracy, and the harmonic mean **H** of base and new classes.

In the few-shot experiment, we compared with Linear Probe, CoOp (Zhou et al., 2022b), and ProGrad (Zhu et al., 2023) using ResNet50 as backbone, while in the base-to-new generalization experiment, we compare with CoOp (Zhou et al., 2022b), CoCoOp (Zhou et al., 2022a), KgCoOp (Yao et al., 2023) and ProGrad (Zhu et al., 2023) using ViT-B/16 as backbone. Our selection of comparison methods for few-shot learning was based on several factors: (1) ResNet50 is commonly used as the standard backbone for few-shot learning comparisons, and these methods only provide complete results with ResNet50 backbone, (2) approaches that focus specifically on text prompt optimization (as opposed to visual prompt methods). We note that the research community has recently shifted focus more toward base-to-new generalization scenarios rather than few-shot learning, which is why our few-shot experiments are more targeted. We chose not to include general few-shot learning methods that don't specifically target prompt learning (e.g., prototypical networks, matching networks) as they represent different paradigms and would not provide a fair comparison with our prompt-based approach.

Note that CoCoOp (Zhou et al., 2022a) is instance-conditioned, while other methods are textual-only methods. Textual-only methods typically have poorer generalization to unseen classes within the same task, even lagging behind the original CLIP on some datasets. Instance-conditioned methods improve the generalization by generating different contexts based on various image visual features, and then obtain different text features through the CLIP text encoder. Therefore, they require significant computational resources. Our method, MoCoOp, also partially relies on visual information but does not generate new contexts. Instead, it combines different text features for different images, thus eliminating the heavy computational cost of the text encoder during inference.

Following the previous baselines, we primarily evaluate the accuracy of our approach across a total of 11 datasets. The datasets used include: ImageNet (Deng et al., 2009), Caltech101 (Fei-Fei et al., 2004), Oxford-Pets (Parkhi et al., 2012), Stanford Cars (Krause et al., 2013), Flowers102 (Nilsback and Zisserman, 2008), Food101 (Bossard et al., 2014), FGVC Aircraft (Maji et al., 2013), SUN397 (Xiao et al., 2010), DTD (Cimpoi et al., 2014), EuroSAT (Helber et al., 2019), and UCF-101 (Soomro et al., 2012).

### 3.4 Main results

#### 3.4.1 Results of few-shot experiment

**Figure 3:**
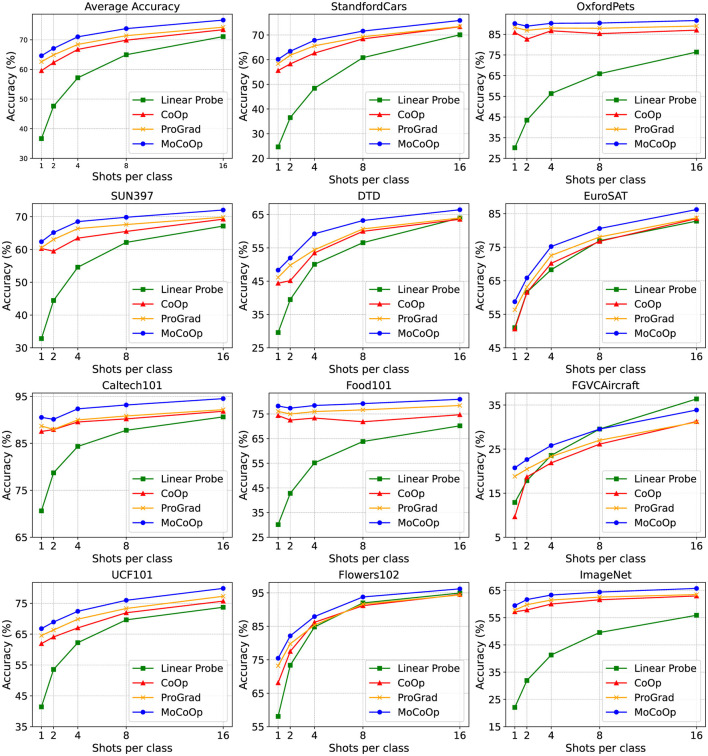
The few-shot learning results on 11 datasets. We plot the results across 1,2,4,8,16 shots. It can be seen that our MoCoOp consistently and significantly surpasses CoOp (Zhou et al., 2022b), ProGrad (Zhu et al., 2023), and the Linear Probe approach across most datasets. This is evident in the average accuracy displayed in the top left corner.

In the Figure 3, we plot the performance curves of our MoCoOp and the baselines across 11 datasets for various shots, along with the average accuracies of all datasets. The results show that MoCoOp consistently outperforms the other methods, particularly in low-shot scenarios (1–2 shots), where its advantage is most pronounced on challenging datasets like FGVC Aircraft, UCF101, and DTD. As the number of shots increases, MoCoOp continues to maintain higher accuracy across all datasets, with the performance gap narrowing slightly at higher shot counts. These results highlight MoCoOp's superior generalization ability and robustness, making it effective in diverse few-shot learning tasks.

#### 3.4.2 Results of base-to-new generalization

In the Table 1, we list the comparison results of MoCoOp and several baselines. The best results are marked in bold font.

**Table 1:** Comparison with baselines on base-to-new generalization.

**Dataset**	**CLIP**			**CoOp**			**CoCoOp**			**ProGrad**			**KgCoOp**			**MoCoOp (Ours)**		
	**Base**	**New**	**H**	**Base**	**New**	**H**	**Base**	**New**	**H**	**Base**	**New**	**H**	**Base**	**New**	**H**	**Base**	**New**	**H**
**Average**	69.34	74.22	71.70	82.63	67.99	74.60	80.47	71.69	75.83	82.48	70.75	76.16	80.73	73.60	77.00	**83.56↑**	**76.98↑**	**80.14↑**
ImageNet	72.43	68.14	70.22	76.46	66.31	71.02	75.98	70.43	73.10	77.02	66.66	71.46	75.83	69.96	72.78	76.52	69.20	72.67
Caltech101	96.84	94.00	95.40	98.11	93.52	95.76	97.96	93.81	95.84	98.02	93.89	95.91	97.72	94.39	96.03	98.43	94.87	96.61
OxfordPets	91.17	97.26	94.12	94.24	96.66	95.43	95.20	97.69	96.43	95.07	97.63	96.33	94.65	97.76	96.18	95.69	97.20	96.44
StanfordCars	63.37	74.89	68.65	76.20	69.14	72.49	70.49	73.59	72.01	77.68	68.63	72.88	71.76	75.04	73.36	76.34	73.26	74.77
Flowers102	72.08	77.80	74.83	97.63	69.55	81.23	94.87	71.75	81.71	95.54	71.87	82.03	95.00	74.73	83.65	97.18	77.21	86.05
Food101	90.10	91.22	90.66	89.44	87.50	88.46	90.70	91.29	90.99	90.37	89.59	89.98	90.50	91.70	91.09	90.62	91.61	91.11
FGVCAircraft	27.19	36.29	31.09	39.24	30.49	34.30	33.41	23.71	27.74	40.54	27.57	32.82	36.21	33.55	34.83	39.67	37.25	38.42
SUN397	69.36	75.35	72.23	80.85	68.34	74.07	79.74	76.86	78.27	81.26	74.17	77.55	80.29	76.53	78.36	81.57	78.08	79.79
DTD	53.24	59.90	56.37	80.17	47.54	59.68	77.01	56.00	64.85	77.35	52.35	62.45	77.55	54.99	64.35	83.10	63.60	72.05
EuroSAT	56.48	64.05	60.03	91.54	54.44	68.27	87.49	60.04	71.21	90.11	60.89	72.67	85.64	64.34	73.48	94.79	85.18	89.73
UCF101	70.53	77.50	73.85	85.14	64.47	73.37	82.33	73.45	77.64	84.33	73.94	79.35	82.89	76.67	79.65	85.28	79.31	82.17

It shows that MoCoOp (Ours) achieves strong performance in base-to-new generalization across 11 datasets, with the highest average Base accuracy (83.56%), New accuracy (76.98%), and Harmonic mean (H) (80.14%). Compared to other methods like CoOp (Zhou et al., 2022b), CoCoOp (Zhou et al., 2022a), ProGrad (Zhu et al., 2023) and KgCoOp (Yao et al., 2023), MoCoOp demonstrates consistent improvements, particularly in achieving a better balance between Base and New class performance. This indicates that MoCoOp is effective in handling both seen and unseen classes across a variety of datasets.

### 3.5 Ablation results

#### 3.5.1 Component analysis

Table 2 presents the performance as we progressively include components. Our baseline is CoOp (Zhou et al., 2022b). As can be seen in Table 2, adding MoE alone has already achieved significant improvement. Adding hard prompt guided routing provides a slight improvement, while incorporating semantically grouped text supervision brings a huge enhancement.

**Table 2:** Impact of the number of selected experts (K) on base-to-new generalization performance.

**Dataset**	**Caltech101**			**EuroSAT**			**UCF101**			**Flowers102**		
	**Base**	**New**	**H**	**Base**	**New**	**H**	**Base**	**New**	**H**	**Base**	**New**	**H**
Baseline	95.40	98.11	93.52	91.54	54.44	68.15	85.14	64.47	73.57	97.63	69.55	81.35
+ MoE	98.38	92.03	95.10	94.90	58.79	72.60	85.78	69.50	76.79	97.63	70.64	81.97
+*L*_router_	98.39	92.47	95.34	95.17	57.05	71.34	86.97	73.88	79.89	97.34	72.77	83.28
+*L*_text_	98.43	94.87	96.61	94.79	85.18	89.73	85.28	79.31	82.17	97.18	77.21	86.05

We sequentially add the components MoE, *L*_router_ and *L*_text_. Our baseline is CoOp (Zhou et al., 2022b).

#### 3.5.2 The number of experts selected by the router

We also show the effect of the number of experts selected on the performance. As seen in Table 3, the results indicate that using the top 2 experts (K = 2) generally achieves the best balance between Base and New accuracy, reflected in the highest Harmonic mean (H) in most cases. While increasing K to 3 or 4 sometimes improves Base accuracy, it often reduces New accuracy and H, suggesting that selecting too many experts may dilute performance on unseen classes. Overall, selecting the top 2 experts provides the best trade-off between Base and New class generalization.

**Table 3:** Comparing randomly grouped hard templates and semantically grouped hard templates.

**Dataset**	**K** = **2**			**K** = **3**			**K** = **4**		
	**Base**	**New**	**H**	**Base**	**New**	**H**	**Base**	**New**	**H**
Caltech101	**98.43**	**94.87**	**96.61**	98.39	94.43	96.37	98.00	94.87	96.41
EuroSAT	**94.48**	**77.02**	**84.75**	94.38	75.0	83.58	94.02	74.36	83.04
UCF101	**85.28**	**79.31**	**82.17**	84.23	68.63	75.63	86.40	79.23	82.66
Flowers102	97.18	**77.21**	**86.05**	**98.10**	71.42	82.66	97.34	76.67	85.78

Bold values indicate the best performance for each metric.

#### 3.5.3 Randomly grouped vs. semantically grouped templates

We compare randomly grouped hard prompt templates and semantically grouped templates, It can be seen in Table 4, that using semantically grouped hard prompt templates consistently improves performance compared to randomly grouped templates across the four selected datasets. The Harmonic mean (H), which balances Base and New accuracy, shows consistent gains with semantic grouping. These results indicate that semantically grouped templates better capture meaningful relationships, enhancing generalization performance across both seen and unseen classes.

**Table 4:** Comparing randomly grouped hard templates and semantically grouped hard templates.

**Dataset**	**Template**	**Base**	**New**	**H**
**Caltech101**	Random	98.31	93.56	95.88
	Semantic	**98.43**	**94.87**	**96.61**
**EuroSAT**	Random	94.78	79.77	86.63
	Semantic	**94.79**	**85.18**	**89.73**
**UCF101**	Random	83.26	77.25	80.14
	Semantic	**85.28**	**79.31**	**82.17**
**Flowers102**	Random	**97.53**	75.95	85.40
	Semantic	97.18	**77.21**	**86.05**

Bold values indicate the best performance for each metric.

#### 3.5.4 Routing regularization loss function

We also compared different routing regularization loss functions. As shown in Table 5, using KL divergence as the loss function generally performs better than using Mean Squared Error (MSE). This may be because KL divergence is more sensitive to distribution differences and can more effectively measure the discrepancy between the predicted distribution and the target distribution. In routing regularization, the model's output often involves probability distributions, and KL divergence is specifically designed to optimize the similarity between distributions, making it more suitable for this scenario. In contrast, MSE only penalizes the squared numerical error, aiming to minimize the absolute difference between the predicted and target values, but it may fail to capture the nuances of probability distributions.

**Table 5:** Ablation study on routing regularization loss functions.

**Dataset**	**Loss function**	**Base**	**New**	**H**
**Caltech101**	MSE	98.39	94.10	96.20
	KL	**98.43**	**94.87**	**96.61**
**EuroSAT**	MSE	94.17	65.64	77.36
	KL	**94.79**	**85.18**	**89.73**
**UCF101**	MSE	**85.37**	77.66	81.33
	KL	85.28	**79.31**	**82.17**
**Flowers102**	MSE	96.87	76.60	85.55
	KL	**97.18**	**77.21**	**86.05**

Bold values indicate the best performance for each metric.

### 3.6 Comparison of inference time

The key differences between CoCoOp and our MoCoOp approach span conceptual design and computational efficiency. Conceptually, while CoCoOp generates new context vectors per instance through a meta-network, MoCoOp selects from fixed pre-trained prompt experts using image features. This fundamental difference leads to substantial computational advantages: CoCoOp requires *O*(*N*×*C*) text encoder calls (for *N* images and *C* classes) by generating unique prompts per image, while MoCoOp achieves efficiency through (1) expert selection via a linear router and (2) weighting of pre-computed text features, needing only *E*×*C* initialization calls (for *E* experts). As shown in Table 6 (measured on Caltech101 using an NVIDIA RTX 3090 with batch size 100), this design reduces inference time by 78% compared to CoCoOp (Zhou et al., 2022a), despite a slight increase over single-prompt CoOp (Zhou et al., 2022b). The efficiency gains stem from reusing pre-computed expert prompts rather than generating new ones per instance, making the computational trade-off worthwhile given our performance improvements.

**Table 6:** Inference time comparison on the Caltech101 dataset.

**Method**	**Accuracy**			**Inference time**
	**Base**	**New**	**H**	
CoOp (Zhou et al., 2022b)	98.11	93.52	95.76	0.09s
CoCoOp (Zhou et al., 2022a)	97.96	93.81	95.84	1.14s
**MoCoOp (Ours)**	**98.43**	**94.87**	**96.61**	0.25s

Bold values indicate the best performance for each metric.

### 3.7 Sensitivity to loss weights

**Figure 4:**
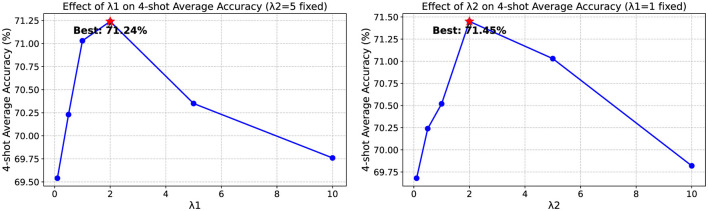
Ablation study on the sensitivity to hyper-parameters λ_1_ and λ_2_.

We conducted experiments to analyze the sensitivity of our model to the two loss weights: λ_1_, which controls the weight of the router regularization loss, and λ_2_, which regulates the text-level supervision. As shown in Figure 4, we varied each parameter while keeping the other fixed at its default value. The results indicate that both parameters significantly influence model performance, with λ_1_ primarily affecting the router's ability to select appropriate prompts, and λ_2_ impacting the preservation of knowledge from hard prompts. We observe that optimal performance is generally achieved when λ_1_ is set between 0.5 and 1.5, and λ_2_ between 4.0 and 6.0. Values outside these ranges tend to either insufficiently constrain the model or overly restrict its adaptability. This demonstrates the importance of balancing these regularization terms for effective prompt learning.

### 3.8 Impact of the total expert number

We further investigate how the total number of experts impacts model performance on the base-to-new generalization task. By default, our model uses 5 experts for Caltech101 and Flowers102, and 6 experts for EuroSAT and UCF101. To evaluate the influence of expert count, we conducted experiments with varying numbers of experts (from 3 to 8) while keeping other hyperparameters fixed. We use GPT-4 (Achiam et al., 2023) to generate the hard prompt templates.

As shown in Table 7, the optimal number of experts tends to align with our default configurations, though the performance differences across different expert counts are relatively small. For Caltech101 and Flowers102, 5 experts yield slightly better performance, while EuroSAT and UCF101 benefit marginally from 6 experts. Interestingly, using fewer experts (e.g., 3) still produces competitive results, particularly for Flowers102 and Caltech101, suggesting that even a small number of experts can effectively capture diverse image contexts for certain datasets.

**Table 7:** Impact of expert numbers on base-to-new generalization performance.

**Dataset**	**Metric**	**3 Experts**	**5 Experts**	**6 Experts**	**8 Experts**
**Caltech101**	Base	97.85	**98.43**	98.29	98.32
	New	93.95	**94.87**	94.32	94.76
	H	95.86	**96.61**	96.26	96.50
**EuroSAT**	Base	93.24	94.58	**94.79**	94.63
	New	79.36	83.42	**85.18**	84.73
	H	85.72	88.67	**89.73**	89.40
**UCF101**	Base	84.15	85.18	**85.28**	85.04
	New	76.87	78.95	**79.31**	78.82
	H	80.35	81.97	**82.17**	81.80
**Flowers102**	Base	96.92	**97.18**	97.05	96.83
	New	76.94	**77.21**	77.06	76.58
	H	85.86	**86.05**	85.92	85.54

Bold values indicate the best performance for each metric.

These results indicate that while our default configurations generally work well, the model is somewhat robust to the choice of expert count. The slight differences in performance suggest that the ideal number of experts may vary based on dataset characteristics, but not dramatically so. This relative stability across different expert counts highlights the adaptability of our mixture-of-experts approach, which can perform effectively across a range of configurations.

### 3.9 Qualitative analysis

**Figure 5:**
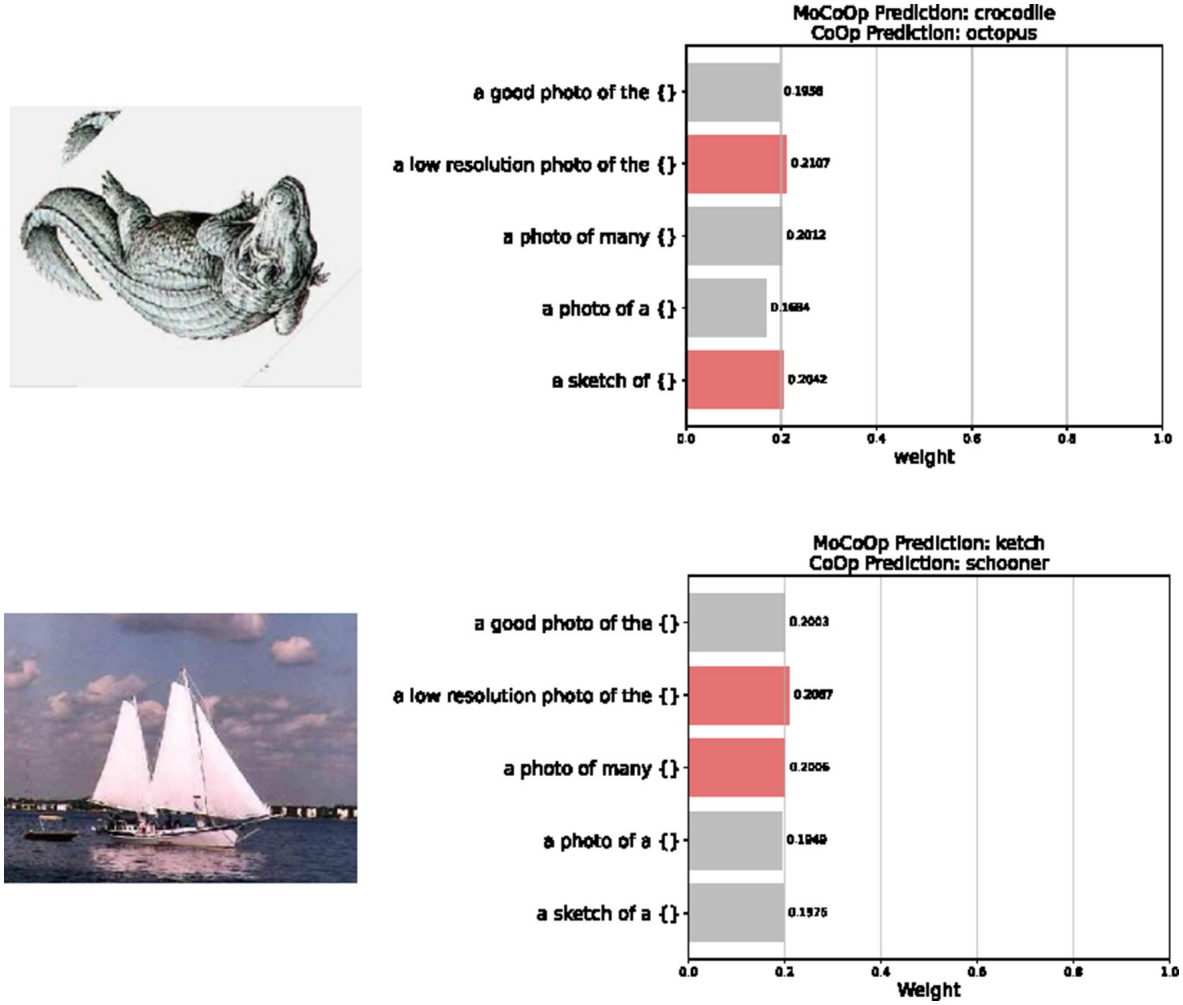
Visualization of prompt selection across different image samples. The router dynamically selects the most suitable prompts based on the visual content of each image, while the traditional method, such as the CoOp gives the wrong answer.

As shown in Figure 5, our MoCoOp model dynamically selects the most appropriate prompt templates based on image content features. For example, in the first image of a crocodile sketch from the Caltech101 dataset (Fei-Fei et al., 2004), the router recognizes the artistic nature of the image, assigning higher weights to the “a low resolution photo of the {}” template (0.21) and the “a sketch of {}” template (0.20). This allows MoCoOp to correctly classify the image as a “crocodile,” while CoOp incorrectly labels it as “octopus.” Similarly, for the second image of a sailing vessel from the same dataset, the model activates the “a low resolution photo of the {}” template (0.21) along with the “a photo of many {}” template (0.20), correctly identifying the vessel as a “ketch” where CoOp mislabels it as “schooner.” This demonstrates that our routing mechanism effectively captures the stylistic and semantic content of images and associates them with the most relevant textual descriptions. By activating different template combinations for different types of images, MoCoOp achieves greater adaptability and robustness compared to single-prompt learning methods. The router not only learns how to select appropriate prompts but also how to allocate weights to reflect the importance of each prompt for specific visual characteristics.

## 4 Conclusion

In this work, we introduce a novel mixture-of-prompts learning method for vision-language models, addressing key challenges of image context style variations and overfitting. Our approach employs a routing module to dynamically select the most suitable prompts(styles) for each instance, enhancing adaptability and performance. We also propose a hard prompt guided gating loss and semantically grouped text-level supervision, which help maintain initial knowledge and mitigate overfitting. Our method demonstrate improvements across multiple datasets in few-shot learning, and base-to-new generalization scenarios. On the other hand, several aspects of this work warrant further research and optimization in the future. First, while MoCoOp's computational overhead is significantly lower than instance-conditioned approaches like CoCoOp, it remains higher than single-prompt methods. Future work will address this computational cost reduction. Then, manual grouping of prompts requires domain expertise, which may limit easy application to entirely new domains without prior knowledge. LLMs could be used for generating and grouping hard prompt templates in the future. Third, our method is more sensitive to hyperparameter tuning than simpler approaches, particularly regarding the balance between different loss components. Last, future work could also explore extending this methodology to include vision prompts or instance-conditioned contexts for further enhancements.

## Data Availability

The datasets presented in this study can be found in online repositories. The names of the repository/repositories and accession number(s) can be found in the article/Supplementary material.
