# Supplementary material for: Mixture of prompts learning for vision-language models
Source: Front Artif Intell. 2025 Jun 10;8:1580973. doi: 10.3389/frai.2025.1580973 (PMC12185420; doi:10.3389/frai.2025.1580973)
Supplement: Supplementary file 1 [file Data_Sheet_1.pdf]

frontiers

Supplementary Material

1 HARD PROMPT TEMPLATES

Here is the groups of hard prompt templates for each dataset.

1.1 ImageNet

```
[
 # Photos of flowers
 "a photo of a {}, a type of flower.",
 # Photos of people doing activities
 "a photo of a person doing {}.",
 # Satellite photos
 "a centered satellite photo of {}.",
 # Photos of aircraft
 "a photo of a {}, a type of aircraft.",
 # "Itap" (I took a picture) photos
 "itap of a {}.",
 "itap of the {}.",
 # Photos of large objects
 "a photo of the large {}.",
 "a photo of a large {}.",
 # Art and renditions
 "art of the {}.",
 "a rendering of a {}.",
 "a rendering of the {}.",
 "a rendition of the {}.",
 # Photos of small objects
 "a photo of the small {}.",
 # General photo prompts
 "a photo of a {}.",
 "a photo of the {}.",
 "a photo of many {}.",
 # Low resolution and pixelated photos
 "a low resolution photo of the {}.",
 "a low resolution photo of a {}.",
 "a pixelated photo of the {}.",
 "a pixelated photo of a {}.",
 "a jpeg corrupted photo of the {}.",
 "a blurry photo of a {}.",
 "a bad photo of the {}.",
 # Cropped photos
 "a cropped photo of the {}.",
 "a cropped photo of a {}.",
```

```
    # Bright photos
    "a bright photo of the {}.",
    # Good quality photos
    "a good photo of the {}.",
    "a good photo of a {}.",
    # Close-up photos
    "a close-up photo of the {}.",
    # Jpeg corrupted photos
    # Blurry photos
    # Clean objects
    "a photo of the clean {}.",
    # Video game screenshots
    "a {} in a video game.",
    # Hard to see objects
    "a photo of the hard to see {}.",
    # Bad quality photos
    # Origami photos
    "a origami {}.",
    # Texture photos
    "{} texture.",
    ]
```

1.2 Caltech101

```
[
    "{} texture.",
    # General photo prompts
    "a photo of a {}.",
    "a photo of the {}.",
    "a photo of many {}.",
    # Low resolution and pixelated photos
    "a low resolution photo of the {}.",
    "a low resolution photo of a {}.",
    "a pixelated photo of the {}.",
    "a pixelated photo of a {}.",
    # Good quality photos
    "a good photo of the {}.",
    "a good photo of a {}.",
 ]
```

1.3 DTD

```
[
        "{} texture.",
    ## General photo prompts
    "a photo of a {}.",
```

```
    "a photo of the {}.",
    "a photo of many {}.",
    # Low resolution and pixelated photos
    "a low resolution photo of the {}.",
    "a low resolution photo of a {}.",
    "a pixelated photo of the {}.",
    "a pixelated photo of a {}.",
    # Good quality photos
    "a good photo of the {}.",
    "a good photo of a {}.",
    ]
```

1.4 EuroSAT

```
[
    # Photos of aircraft
    "a photo of a {}, a type of aircraft.",
    "a centered satellite photo of {}.",
    # General photo prompts
    "a photo of a {}.",
    "a photo of the {}.",
    #
    "a photo of the large {}.",
    "a photo of a large {}.",
    #
    "a photo of the small {}.",
    "a photo of a small {}.",
    # Low resolution and pixelated photos
    "a low resolution photo of the {}.",
    "a low resolution photo of a {}.",
    "a pixelated photo of the {}.",
    "a pixelated photo of a {}.",
    # Good quality photos
    "a good photo of the {}.",
    "a good photo of a {}.",
    ]
```

2 FGVCAIRCRAFT

```
[
    # Photos of aircraft
    "a photo of a {}, a type of aircraft.",
    # General photo prompts
    "a photo of a {}.",
    "a photo of the {}.",
    "a photo of many {}.",
```

```
    # Low resolution and pixelated photos
    "a low resolution photo of the {}.",
    "a low resolution photo of a {}.",
    "a pixelated photo of the {}.",
    "a pixelated photo of a {}.",
    # Good quality photos
    "a good photo of the {}.",
    "a good photo of a {}.",
    ]
```

3 OXFORDFLOWERS

```
 [
    # Photos of flowers
      "a photo of a {}, a type of flower.",
    # General photo prompts
        "a photo of a {}.",
        "a photo of the {}.",
        "a photo of many {}.",
    # Low resolution and pixelated photos
        "a low resolution photo of the {}.",
        "a low resolution photo of a {}.",
        "a pixelated photo of the {}.",
        "a pixelated photo of a {}.",
    # Good quality photos
        "a good photo of the {}.",
        "a good photo of a {}.",
    ]
```

4 OXFORDPETS

```
[
        "a photo of the small {}.",
    #
        "a photo of the large {}.",
        "a photo of a large {}.",
    # General photo prompts
        "a photo of a {}.",
        "a photo of the {}.",
        "a photo of many {}.",
    # Low resolution and pixelated photos
        "a low resolution photo of the {}.",
        "a low resolution photo of a {}.",
        "a pixelated photo of the {}.",
        "a pixelated photo of a {}.",
```

```
    # Good quality photos
        "a good photo of the {}.",
        "a good photo of a {}.",
    ]
```

5 FOOD101

```
[
    # General photo prompts
        "a photo of a  {}.",
    #
        "a photo of the delicious {}.",
        "a photo of the tasty {}.",
        "A dish of the {}.",
    # Low resolution and pixelated photos
        "a low resolution photo of the {}.",
        "a low resolution photo of a {}.",
        "a pixelated photo of the {}.",
        "a pixelated photo of a {}.",
    # Good quality photos
        "a good photo of the {}.",
        "a good photo of a {}.",
    ]
```

6 STANFORDCARS

```
[
        "A photo of the high-performance {}.",
        "A photo of the modern {}.",
        "A photo of the fast {}.",
    # General photo prompts
        "a photo of a {}.",
        "a photo of the {}.",
        "a photo of many {}.",
    # Low resolution and pixelated photos
        "a low resolution photo of the {}.",
        "a low resolution photo of a {}.",
        "a pixelated photo of the {}.",
        "a pixelated photo of a {}.",
    # Good quality photos
        "a good photo of the {}.",
        "a good photo of a {}.",
    ]
```

7 SUN397

```
[
    # General photo prompts
        "a photo of a {}.",
        "a photo of the {}.",
        "a photo of many {}.",
    # Low resolution and pixelated photos
        "a low resolution photo of the {}.",
        "a low resolution photo of a {}.",
        "a pixelated photo of the {}.",
        "a pixelated photo of a {}.",
    # Good quality photos
        "a good photo of the {}.",
        "a good photo of a {}.",
    ]
```

8 UCF101

```
  [
        "a photo of a person doing {}.",
    #
        "a {} in a video game.",
    # General photo prompts
        "a photo of a {}.",
        "a photo of the {}.",
        "a photo of many {}.",
    ## Low resolution and pixelated photos
        "a low resolution photo of the {}.",
        "a low resolution photo of a {}.",
        "a pixelated photo of the {}.",
        "a pixelated photo of a {}.",
    ## Good quality photos
        "a good photo of the {}.",
        "a good photo of a {}.",
    ]
```
